# DISTANCE FROM APARTMENT TO ACTIVITY AS A BARRIER TO PARTICIPATION IN GROUP ACTIVITIES FOR ASSISTED LIVING RESIDENTS

**DOI:** 10.1093/geroni/igz038.934

**Published:** 2019-11-08

**Authors:** Rebecca L Mauldin, Jason Fernandez

**Affiliations:** 1 University of Texas at Arlington, Arlington, Texas, United States; 2 University of Houston, Houston, Texas, United States

## Abstract

Participating in group activities is potentially also beneficial for residents of assisted living facilities, who have decreased independence due to physical or cognitive limitations. However, their impaired abilities can make attending group activities difficult. One potential barrier is the physical distance from residents’ apartments to the activity location. This study analyzed resident (N=30) attendance at group activities in an assisted living facility. Attendance records for 822 group activities over the course of six months and the distance between residents’ apartments and activity location were analyzed. Exponential random graph models were used to estimate the effect of individual-level, activity-level, and social network factors on the likelihood that a resident attends an activity. On average, residents attended 128 events (SD = 151) during the six month study period. The average size of activities was 4.6 residents (SD = 2.9). The closest distance between a resident’s apartment and an activity was 7.7 feet; however, some residents had to travel up to 382 feet to attend an activity (M=153 ft.; SD=52). After controlling for individual-, activity-, and social network-level factors, distance to the activity was significantly and negatively associated with the likelihood a resident attended the activity. In spite of the fact that assisted living facilities offer supports and services to help residents overcome their physical limitations, it appears that distances within the building can be barriers to participation in group activities. This should be taken into account in designing facilities, locating group activities, and devising strategies to increase participation in group activities.

